# Bronchial provocation tests in clinical practice

**DOI:** 10.1590/S1516-31802011000400008

**Published:** 2011-05-05

**Authors:** Marcos de Carvalho Borges, Erica Ferraz, Elcio Oliveira Vianna

**Affiliations:** I MD, PhD. Visiting Professor, Department of Medicine, Faculdade de Medicina de Ribeirão Preto (FMRP), Ribeirão Preto, São Paulo, and Adjunct Professor, Department of Medicine, Universidade de São Carlos (UFSCar), São Carlos, São Paulo, Brazil.; II PhD. Research Collaborator, Department of Medicine, Faculdade de Medicina de Ribeirão Preto (FMRP), Ribeirão Preto, São Paulo, Brazil.; III MD, PhD. Associate Professor, Department of Medicine, Faculdade de Medicina de Ribeirão Preto (FMRP), Ribeirão Preto, São Paulo, Brazil.

**Keywords:** Bronchial provocation test, Asthma, Bronchoconstriction, Diagnosis, Respiratory hypersensitivity, Testes de provocação brônquica, Asma, Broncoconstrição, Diagnóstico, Hipersensibilidade respiratória

## Abstract

Bronchial hyperresponsiveness, which consists of an exaggerated response of the airways to bronchoconstrictor stimuli, is one of the main characteristics of asthma, presented in nearly all asthmatic patients. Bronchial hyperresponsiveness may also be present in other diseases, such as allergic rhinitis, chronic obstructive pulmonary disease, cystic fibrosis, heart failure and respiratory infection, and with some medications, such as β-blockers. Bronchial provocation tests (also known as bronchial challenges) are used to evaluate bronchial responsiveness. These tests have become increasingly used over the last 20 years, with the development and validation of accurate, safe and reproducible tests, and with the publication of well-detailed protocols. Several stimuli can be used in a bronchial challenge, and they are classified as direct and indirect stimuli. There are many indications for a bronchial challenge. In this review, we discuss the main differences between direct and indirect stimuli, and the use of bronchial challenges in clinical practice, especially for confirming diagnoses of asthma, exercise-induced bronchoconstriction and cough-variant asthma, and for use among elite-level athletes.

## RESUMO

Hiperresponsividade brônquica, caracterizada por uma resposta exagerada das vias aéreas a um estímulo broncoconstritor, é uma das principais características da asma, presente em praticamente todos pacientes asmáticos. A hiperresponsividade brônquica pode estar presente também em outras doenças, como rinite alérgica, doença pulmonar obstrutiva crônica, fibrose cística, insuficiência cardíaca, infecção respiratória e com o uso de algumas medicações, como β-bloqueadores. Os testes de broncoprovocação são utilizados para determinação da responsividade brônquica, e têm sido cada vez mais utilizados nos últimos 20 anos com o desenvolvimento e validação de testes acurados, seguros e reprodutíveis e com a publicação de protocolos bem detalhados. Diversos estímulos podem ser utilizados em um teste de broncoprovocação, sendo classificados em estímulos diretos e indiretos. Existem inúmeras indicações para um teste de broncoprovocação. Nesta revisão, nós discutimos as principais diferenças dos estímulos diretos e indiretos e o uso desses testes na prática clínica, especialmente para a confirmação de asma, broncoconstrição induzida por exercício, tosse variante de asma e em atletas de elite.

## INTRODUCTION

Bronchial responsiveness is characterized by a change in airway caliber in response to bronchoconstrictor and/or bronchodilator stimuli. Bronchial hyperresponsiveness (BHR) is defined as increased responsiveness in comparison with an expected response. This increased response of the airways can be measured by inhalation of specific or non-specific stimuli, and it should not be expected to be observed in normal subjects.

Bronchial hyperresponsiveness is measured in laboratories by a bronchial provocation test (also known as a bronchial challenge), and is considered present when the dose-response curve for a bronchoconstrictor stimulus displays: a leftward shift (hypersensitivity), an increased slope (hyperreactivity) and an enhanced maximal response compared with the response for a non-asthmatic subject.^1–3^ In other words, BHR is detected by an abnormal response to a bronchoconstrictor stimulus.

Bronchial hyperresponsiveness is one of the main pathophysiological characteristics of asthma and is present in nearly all asthmatic patients, especially during symptomatic episodes.^4^ It can also be found in other diseases, such as allergic rhinitis, chronic obstructive pulmonary disease, cystic fibrosis and heart failure, or after a respiratory infection, and with some medications like β-blockers.^3,4^ Hyperresponsiveness can explain many clinical features of diseases, such as coughing, wheezing, chest tightness or dyspnea, which can occur after allergen or occupational exposure, physical activity, respiratory infections or medications.^3,4^

Although bronchial hyperresponsiveness is incompletely understood, it is the ultimate result from an interaction between complex and multiple mechanisms, such as inflammation, alterations in airway smooth muscle, airway remodeling, reduced airway caliber and interaction between airway and lung parenchyma.^5–8^ In this review, we discuss the main differences between direct and indirect stimuli, and the use of bronchial challenges in clinical practice, especially for confirming the diagnoses of asthma, exercise induced bronchoconstriction and cough-variant asthma, and for evaluating dyspnea among elite-level athletes. Systematic reviews and practice guidelines were searched in the Cochrane Library, PubMed, Scopus, Embase, Lilacs, and Medline using the keywords Bronchial Provocation Tests and Bronchial Challenge. Additionally, we included some relevant original studies (Table 1).

**Table 1.** Reviews and practice guidelines were searched in the Cochrane Library, PubMed, Scopus, Embase, Lilacs and Medline databases, using the keywords Bronchial Provocation Tests and Bronchial Challenge. Additionally, relevant original articles were included

Database	Search strategy	Results	
		Found	Used
Cochrane	Bronchial Provocation Tests (MeSH)	0	0
Library	Bronchial Challenge (MeSH)	2	0
PubMed	Bronchial Provocation Tests (MeSH)	585	13
	Bronchial Challenge (MeSH)	49	2
Scopus	Bronchial Provocation Tests (MeSH)	607	14
	Bronchial Challenge (MeSH)	386	13
Embase	Bronchial Provocation Tests (MeSH)	329	3
	Bronchial Challenge (MeSH)	64	4
Lilacs	Bronchial Provocation Tests (MeSH)	1	0
	Bronchial Challenge (MeSH)	1	0
Medline	Bronchial Challenge (MeSH)	88	1
	Bronchial Provocation Tests (MeSH)	98	1
Description of articles used in our review	Review		20
	Practice guidelines		4
	Original studies		25

### Bronchial provocation tests

Generally, bronchial responsiveness is measured in the laboratory as the change in airway caliber occurring after inhalation of a bronchoconstrictor stimulus.^2^ These tests have become increasingly used over the last 20 years, with the development of accurate, safe and reproducible bronchial challenges, and with the publication of well-detailed protocols. Bronchial challenge tests are easily performed in adults and children more than seven years of age.^3,6,9^

**Figure 1:**
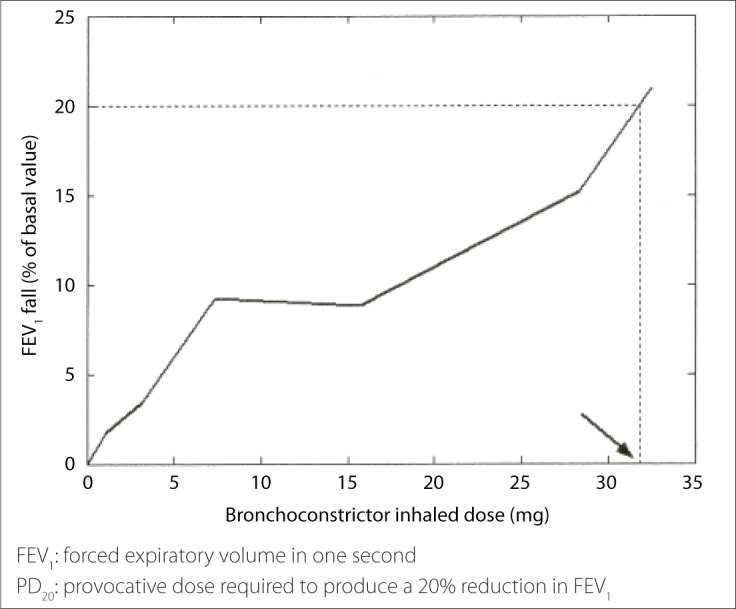
Example of PD_20_ calculation for hypertonic saline bronchial challenge obtained by interpolation between the last two points (second-to-last and final concentration) of the dose-response curve.

Bronchial challenges are performed by the inhalation of a bronchoconstrictor stimulus followed by assessment of its response in terms of lung function, especially forced expiratory volume in one second (FEV_1_). When a fall in FEV_1_ is achieved, usually between 10 and 20% from the baseline measurement, the test is stopped and a concentration or dose is calculated and named provocative concentration (PC) or provocative dose (PD), respectively (Figure 1).^3,6,9,10^

The absolute contraindications for bronchial challenges are severe airflow limitation (FEV_1_ < 50% predicted or < 1.0 l), heart attack or stroke within the last three months, uncontrolled hypertension, systolic BP > 200 mmHg, or diastolic BP > 100 mmHg, and known aortic aneurysm. The relative contraindications are moderate airflow limitation (FEV_1_ < 60% predicted or < 1.5 l), inability to perform acceptable-quality spirometry, pregnancy, nursing mothers, current use of cholinesterase inhibitor medication (for myasthenia gravis) and epilepsy requiring medical treatment.^3,6,9,10^

Medications known to influence bronchial responsiveness should be withheld before the test: oral inhaled short-acting (e.g. salbutamol, terbutaline or fenoterol), medium-acting (e.g. ipratropium or theophylline) and long-acting (e.g. formoterol, tiotropium or salmeterol) bronchodilators should be withheld for 8, 24, and 48 hours, respectively; leukotriene modifiers for 24 hours; cromolyn sodium for 8 hours; nedocromil for 48 hours; and, except for methacholine challenge, antihistamines are withheld for their duration of action. Coffee, tea, cola drinks and chocolate should not be consumed on the day of the test. The intervals between exposures and tests should be taken into account. In order to avoid effects on the results, intervals should be respected, as follows: one to three weeks after environmental antigens, three to six weeks after respiratory infections, months after occupational sensitizers, one week after air pollutants and days to months after chemical irritants.^3,6,9,10^

Bronchial challenges should be performed in the presence of staff with appropriate training for treating acute bronchoconstriction, including appropriate use of resuscitation equipment, which must be close enough to respond quickly to an emergency. Medications to treat severe bronchoconstriction must be present in the room where the test takes place. They include epinephrine and atropine for subcutaneous injection, and salbutamol/albuterol and ipratropium in metered-dose inhalers or premixed solutions for inhalation. Oxygen must be readily available. A small-volume nebulizer should be readily available for administration of bronchodilators. A stethoscope, sphygmomanometer and pulse oximeter should be available. Patients should not be left unattended during the procedure. Usually, the bronchoconstriction is transitory and easily managed with bronchodilators. The room needs to be efficiently ventilated, and staff with active asthma should not perform the test or be present in the room where it takes place.^3,6,9,10^

### Direct and indirect stimuli

Several stimuli are in use for bronchial challenges, and each may constitute a piece of the jigsaw puzzle on the pathophysiology of BHR and respiratory diseases.^11^ The bronchial stimuli can be classified into direct and indirect stimuli, according to the main/dominant mechanism through which they cause the bronchoconstriction.^12^ This classification highlights the heterogeneity of the airway response to the different stimuli, and helps in understanding the differences in sensitivity and specificity of the stimuli and the effect of treatment on hyperresponsiveness.^5,10,12^ Therefore, the results from different bronchial challenges should be interpreted according to the stimulus used and the disease phase and treatment.

Direct stimuli cause bronchoconstriction acting on the effector cells, such as airway smooth muscle cells, bronchial vascular endothelial cells and mucus-producing cells.^5^ The main direct stimulus used in clinical practice is methacholine. Indirect stimuli cause bronchoconstriction through action on another cells, such as inflammatory cells, epithelial cells and/or neuronal cells, which interact secondarily with these effector cells.^5,11^ The main indirect stimuli used in clinical practice are hypertonic saline, mannitol, bradykinin, exercise and eucapnic voluntary hyperpnea. Some stimuli are able to cause bronchoconstriction through both direct and indirect action. Several stimuli used for bronchial challenge are listed in Chart 1.

**Chart 1:** Direct and indirect stimuli used to measure bronchial responsiveness. Direct stimuli cause bronchoconstriction through action on effector cells and indirect stimuli cause bronchoconstriction through action on another cells, which interact secondarily with the effector cells. Some stimuli are able to cause bronchoconstriction through both direct and indirect action. The stimuli are classified according their main/dominant mechanism

Direct stimuli	
	Methacholine
	Histamine
	Acetylcholine
	Carbachol
	Prostaglandin D2
	Leukotriene C4/D4/E4
**Indirect stimuli**	
	Hypertonic aerosols (hypertonic saline, mannitol)
	Hypotonic aerosols
	Exercise
	Eucapnic voluntary hyperpnea
	Bradykinin
	Adenosine
	Propranolol
	Metabisulphite
	Tachykinins

Both direct and indirect stimuli can be used as complementary information in diagnosing respiratory diseases, such as asthma, cough and exercise-induced bronchoconstriction (EIB). Although there are differences in the airway response to the different stimuli, our experience shows that it is more important, in general practice, to get used to one bronchoconstrictor stimulus and protocol (thus knowing its advantages and limitations), than to use several stimuli only few times each (thus knowing several stimuli superficially).

The major difference between direct and indirect stimuli, in general practice, is the sensitivity and specificity of each stimulus in diagnosing respiratory diseases, especially asthma. Bronchial challenge with direct stimuli, such as histamine and methacholine, is extremely sensitive for diagnosing asthma patients. However, these stimuli lack specificity, both in differentiating asthma from normal and asthma from chronic airflow limitation.^4^ Therefore, while a positive challenge in a symptomatic patient, especially with direct stimuli, does not confirm asthma, a negative test has a highly negative predictive value and rules out asthma.

Indirect stimuli are used in bronchial challenges because of their higher specificity for identifying people with active asthma, and to evaluate treatments with anti-inflammatory drugs.^5,10,12^ The major advantage of indirect stimuli is their capacity to act on many different cells, so that a wide variety of substances contribute towards the airway narrowing (e.g., histamine, leukotrienes, prostaglandins and neuropeptides), which makes the response more similar to bronchoconstriction developed during daily activities.^10,13,14^ Since indirect stimuli have higher specificity, another advantage is the lack of false-positive tests; hence the interest in these stimuli in epidemiological studies on asthma prevalence.^10,15^ It has been suggested that indirect stimuli would better reflect the degree of airway inflammation than would direct stimuli, with greater usefulness for evaluating and monitoring the response to anti-inflammatory drugs.^5,16,17^

### The use of bronchial provocation tests in general practice

There are many indications for a bronchial challenge, such as to confirm the diagnosis of asthma and/or EIB; to investigate cough-variant asthma; to investigate occupational asthma; to identify and evaluate the response and efficacy of drugs, like anti-inflammatory drugs; to collect sputum; to identify those who may experience airway narrowing while diving with self-contained underwater breathing apparatus (SCUBA); to clear unwanted secretions; to differentiate asthma from chronic airflow limitation; and to evaluate elite-level athletes.^4,10^ (Chart 2).

**Chart 2:** Indications for a bronchial challenge

To confirm the diagnosis of asthma
To confirm the diagnosis of exercise-induced bronchoconstriction
To investigate cough-variant asthma
To identify and evaluate the response and efficacy of drugs, like anti-inflammatory drugs
To collect sputum
To identify those who may experience airway narrowing while diving with self-contained underwater breathing apparatus (SCUBA)
To clear unwanted secretions
To differentiate asthma from chronic airflow limitation
To evaluate elite-level athletes

Despite the numerous indications, in general practice, bronchial challenges are mostly used for confirming the diagnoses of asthma, EIB and cough-variant asthma, and for assessing elite-level athletes, as will be discussed subsequently.^4,10^

### The use of bronchial provocation tests in diagnosing asthma

A correct diagnosis for asthma is essential for adequate therapy. Asthma generally manifests with symptoms of episodic breathlessness, wheezing, coughing and chest tightness and is confirmed through airflow limitation and reversibility, as detected through lung function tests (spirometry or peak expiratory flow).^18^ Other tests, such as measurements of allergic status, airway responsiveness and inflammation, can be used in atypical presentations.^18^

Among patients with symptoms consistent with asthma who present normal lung function, an objective measurement such as a bronchial challenge, with direct or indirect stimuli, may help establish a diagnosis of asthma.^10,19,20^ In these patients, detection of BHR suggests the diagnosis of asthma. The level of BHR in the bronchial challenge can also be used. Although there is no threshold that differentiates asthma from other diseases that present BHR, the BHR of asthma patients is usually more severe. Therefore, there is a correlation between the degree of airway responsiveness and asthma symptoms,^21,22^ e.g. a histamine or methacholine PC_20_ greater than 8 (or 16) mg/ml rules out current asthma in most instances, whereas a PC_20_ less than 1 mg/ml is almost diagnostic of current asthma. Values between 1 and 8 mg/ml are intermediate in this regard.^23^ Since bronchial challenges have high sensitivity and limited specificity, a negative result can be useful for ruling out a diagnosis of persistent asthma, but a positive result does not always confirm the diagnosis.^18,21,23^ If the diagnosis after the bronchial challenge results remains uncertain, other tests, such as prick test, IgE measurement or induced sputum can be used as measurements of allergic status and airway inflammation, and/or a therapeutic test could be initiated.

It is important to evaluate the patient's clinical status and the medications used, given that the BHR is a dynamic process that can vary over time. It can increase after exposure to various environmental sensitizers and drugs (e.g. airborne allergens, substances found at the workplace, respiratory infections or propranolol), and it can decrease spontaneously or after anti-inflammatory therapy.^21,24,25^

### The use of bronchial provocation tests in diagnosing cough-variant asthma

Asthma is one of the most common etiologies among patients with a chronic cough. Other common diagnoses are postnasal drip syndrome and gastroesophageal reflux.^26,27^ Some patients with asthma have coughing as the sole symptom, and this is termed cough-variant asthma.^28^ Therefore, among patients with a chronic cough, asthma should always be considered as a potential etiology.^26^

Among patients with a chronic cough associated with reversible airway flow obstruction, the diagnosis of asthma should be considered and treatment for asthma should be initiated. However, among patients with a chronic cough and normal spirometry, additional tests should be done, including bronchial challenge. In these patients, the presence of BHR documented in a bronchial challenge suggests the diagnosis of asthma, and treatment should be initiated in order to confirm the diagnosis. Evaluation of treatment response is essential, since the diagnosis of asthma is confirmed only when a positive bronchial challenge is followed by a favorable response to asthma therapy, usually with the use of inhaled corticosteroids for one week.^29^ On the other hand, given the high specificity of bronchial challenges, a negative result rules out asthma from the differential diagnosis of chronic cough.^1^ Methacholine has been the stimulus most frequently indicated and used in cough-variant asthma investigations. Some studies have demonstrated that indirect stimuli could also be used, but more studies are needed before that can be used in routine practice.^30,31^

### Bronchial provocation tests in exercise-induced bronchoconstriction

Exercise-induced bronchoconstriction (EIB) is characterized by an acute, transient airway narrowing that occurs during and most often after exercise.^32^ Common symptoms include coughing, wheezing, chest tightness and dyspnea, usually 5 to 10 min after exercise ceases, and they can remain significant for 30 min if no therapy is provided.^33^ EIB is more frequent after vigorous exercise and in cold and dry weather. EIB is found in 50 to 90% of all asthmatic patients and also occurs in up to 10% of subjects who are not known to be atopic or asthmatic.^34,35^ The prevalence of EIB among athletes is higher, and notifications by athletes have been increasing in recent Olympic Games.^32,36^ Because athletes without asthma may have been using inhaled β2-agonists in order to improve performance, it has been recommended that athletes should demonstrate current asthma, EIB or BHR in order to be approved to inhale β2-agonists at the Olympic Games.^36^

Among patients with a diagnosis of asthma, a complete history associated with therapeutic test is enough for a diagnosis of EIB. Among patients with symptoms of EIB and normal spirometry, and among elite athletes, bronchial challenges are necessary. The most accurate tests for assessing EIB are exercise, eucapnic voluntary hyperpnea, hyperosmolar aerosols such as 4.5% saline, mannitol and methacholine.^32,36^ Because the airway response depends on the intensity of exertion and on environmental variables such as temperature, air humidity and time of the day,^37,38^ it is crucial to know the conditions under which the bronchial challenge was done, in order to make an appropriate interpretation of its results.^39^

For elite athletes, the bronchial challenges accepted are exercise, eucapnic voluntary hyperpnea, hyperosmolar aerosols such as 4.5% saline, mannitol and methacholine. For exercise and eucapnic voluntary hyperpnea, a fall in FEV_1_ of 10% is consistent with EIB; for hypertonic saline and mannitol, a fall in FEV_1_ of 15% is considered to be BHR; and, for methacholine, a fall in FEV_1_ of 20% confirms EIB diagnosis. Bronchial challenges with other stimuli, such as carbachol, histamine or adenosine monophosphate, have not been accepted by the International Olympic Committee's Medical Commission.^36^ Although laboratory-based challenges can be used for identifying EIB, field-based bronchial challenge has been found to be more sensitive.^40^ Moreover, the sensitivity of methacholine for identifying EIB in elite athletes has been reported to be low and less than 40%. Therefore, among elite athletes, a negative methacholine challenge does not completely rule out EIB, and an indirect stimulus can be used for further evaluation.^36,41,42^ Since many other conditions can mimic EIB, a detailed clinical history and physical examination should precede the bronchial challenge.

### Perspectives

Bronchial challenges are useful in studying pathophysiological characteristics of respiratory diseases, such as the relationship between bronchial responsiveness and airway inflammation and remodeling. They could also be used as an index for asthma severity and clinical control. Moreover, bronchial challenges are an essential step in the development of new anti-asthma treatments and can provide key information on the therapeutic potential of these new agents and their anti-inflammatory effects on the airways.^43–45^

Because of the lack of a reference diagnostic test (gold standard), it is difficult to determine the exact sensitivity and specificity of each different stimulus and for each disease. Some studies rely on physician diagnosis as the gold standard, but such assessments are highly subjective.^41^

Several studies have aimed to use bronchial challenge as an additional therapeutic choice. In cystic fibrosis, inhaled mannitol treatment significantly improved lung function.^46^ We have demonstrated that performing two hypertonic saline challenges in the evening attenuated the nocturnal fall in FEV_1_ among asthmatic patients.^47^

Epidemiological studies on asthma have been hampered by lack of consistency in the results between reports. Most definitions of asthma have emphasized variable airflow obstruction and highlighted inflammation as essential elements of the condition. However, a positive BHR has been used as a relative standard criterion of validity. The association between positive BHR and symptoms has been seen as a gold standard definition of asthma and is a matter discussed in recent studies on asthma detection in large populations.^48,49^
